# Whole Proteome-Based Therapeutic Targets Annotation and Designing of Multi-Epitope-Based Vaccines against the Gram-Negative XDR-*Alcaligenes faecalis* Bacterium

**DOI:** 10.3390/vaccines10030462

**Published:** 2022-03-17

**Authors:** Metab Alharbi, Abdulrahman Alshammari, Abdullah F. Alasmari, Saud Alharbi, Muhammad Tahir ul Qamar, Sumra Wajid Abbasi, Bilal Shaker, Sajjad Ahmad

**Affiliations:** 1 Department of Pharmacology and Toxicology, College of Pharmacy, King Saud University, P.O. Box 2455, Riyadh 11451, Saudi Arabia; mesalharbi@ksu.edu.sa (M.A.); abdalshammari@ksu.edu.sa (A.A.); afalasmari@ksu.edu.sa (A.F.A.); 2 Ministry of Health, Riyadh 11451, Saudi Arabia; sau7d@hotmail.com; 3 Department of Bioinformatics and Biotechnology, Government College University, Faisalabad 38000, Pakistan; 4 NUMS Department of Biological Sciences, National University of Medical Sciences, Rawalpindi 46000, Pakistan; sumra.abbasi@numspak.edu.pk; 5 Department of Biomedical Engineering, Chung-Ang University, 84 Heukseok-ro, Dongjak-gu, Seoul 06974, Korea; ch.bilal321@outlook.com; 6 Department of Health and Biological Sciences, Abasyn University, Peshawar 25000, Pakistan

**Keywords:** *Alcaligenes faecalis*, subtractive proteomics, molecular docking, epitopes, in silico cloning

## Abstract

This study involved therapeutic targets mining for the extremely drug-resistant bacterial species called *Alcaligenes faecalis*, which is known to infect humans. The infections caused by this species in different parts of the human body have been linked with a higher degree of resistance to several classes of antibiotics. Meanwhile, alternate therapeutic options are needed to treat these bacterial infections in clinical settings. In the current study, a subtractive proteomics approach was adapted to annotate the whole proteome of *Alcaligenes faecalis* and prioritize target proteins for vaccine-related therapeutics design. This was followed by targeted protein-specific immune epitope prediction and prioritization. The shortlisted epitopes were further subjected to structural design and in silico validation of putative vaccines against *Alcaligenes faecalis*. The final vaccine designs were also evaluated for potential interaction analysis with human TLR-2 through molecular docking. Finally, the putative vaccines were subjected to in silico cloning and immune simulation approaches to ensure the feasibility of the target-specific vaccine constructs in further experimental designs.

## 1. Introduction

*Alcaligenes* is a phylogenetic subgroup of proteobacteria containing diverse species. Among these, *Alcaligenes faecalis* is one of the subspecies known to utilize several aromatic compounds and heavy metals for energy and carbon metabolism. On the other hand, *Alcaligenes faecalis* (hereafter called *A. faecalis*) is also an extremely drug-resistant, nonfermenting opportunistic bacterium that may cause human infections [1]. *A.* *faecalis* is commonly found in water or soil, and it is responsible for several diseases such as bacteremia, urinary tract infections, endocarditis, and pneumonia, all of which have been associated with this bacterium’s infections [2]. There is lack of available antibiotic therapies, and the bacterium has been resistant to several antibiotics, including aminoglycosides, quinolone, and penicillin [3]. This bacterium has been isolated from several clinical materials, including blood, wound discharge, urine, respiratory secretions, and cerebrospinal fluid [2,4]. The infections have also been found to cause foot ulcer in diabetic patients [5]. The optimal temperatures for the growth of *A. faecalis* range from 20 to 37 °C [6]. The molecular and biochemical analysis of several *A. faecalis* strains also demonstrated potential for biosurfactant production [7] and higher capacity of oxidation of arsenic ability [8], and the ability to act as denitrifying organisms [9]. Furthermore, the *A.* *faecalis* strains are also considered valuable sources in bioremediation and biofertilizer and also as biocontrol agents [10,11].

The extremely drug-resistant nature of *A. faecalis* upon susceptibility testing suggests higher resistance (66%) to meropenem, ceftazidime, and imipenem. In contrast, the sensitivity rate of *A. faecalis* was found lower (about 50%) for antibiotics including piperacillin/tazobactam and ciprofloxacin [12]. The production of several types of β-lactamases by the bacterium has been also linked with hydrolyzing activity against many cephalosporins and penicillin [13]. It has also been reported in the human intestinal tract, with varied chances of causing infections and the ability to affect several organs [14]. However, these infections are not predominantly found in newborns; still chloramphenicol has been linked with treatment in the case of 9-day-old premature twin born with meningitis [15]. Similarly, bacteremia-causing infections were observed in three newborn cases with fatal meningitis [16]. Moreover, these infections have been also found in three newborns with diarrhea and grossly bloody stools, which led to one fatal case [17]. One more case in a two-week-old infant has been also associated with bilateral conjunctivitis [18,19].

This bacterium is commonly transmitted through nebulizers and ventilation equipment as droplets. However, infections mediated by direct contact with infected individuals have been also reported, with the potential to cause fatality [20,21]. The infections have been also linked with pan-drug-resistance, causing uncommon bloodstream infections with tigecycline as an effective therapy against it [22,23]. *A. faecalis* has been also known to cause eye infections and peritonitis [24]. Still, the extremely drug-resistant *A. faecalis* [12] also demands the search for advanced therapeutic options that can be utilized to combat this human pathogen. Advanced strategies are also required to depict potential therapeutic targets in the design of alternate anti-*A. faecalis* therapies. Till now, vaccination has been widely utilized to combat against several microbial infections. Such vaccines help to generate adaptive immune responses through the delivery of identified antigenic components to trigger the host immune system against the human pathogens.

Computational methods and resources have been widely deployed in the exploration of biological mechanisms and in the design of therapeutics against human pathogens [25]. This also employs the use of subtractive proteomics techniques to design vaccine therapeutics against several organisms [26]. In several studies, similar in silico techniques were employed in order to propose potent vaccine candidates and to subsequently validate the vaccine candidates experimentally [27,28,29]. In a study, the authors performed a comparative analysis of *Mycobacterium tuberculosis* and identified major and HLA-promiscuous antigenic proteins and peptides which showed strong immunogenicity in in vivo and in vitro analysis [29]. In another study, Liu et al. predicted the MHC class-1 of *Ebolavirus* using in silico approaches, and subsequently, their anti-EBOV immune responses were confirmed by in vivo experiments [27].

In this study, the whole proteome of *A. faecalis* was screened to identify proteins as therapeutic targets that were prioritized based on subcellular localization, non-human homologous nature, and antigenicity potential. The analysis also involved the screening of putative immune epitopes (T-cell, HTL, and B-cell) for the shortlisted target proteins. The highly antigenic and non-allergenic epitopes were then used in the construction of final vaccine designs. Furthermore, topographical organization followed by structural modeling and validations were also performed in this study. The in silico cloning and immune simulation approaches deployed in the study were aimed at providing pre-validated putative vaccine therapeutic options against *A. faecalis.* The experimental processing of the candidate multi-epitope-based vaccine designs contained targeted protein-derived antigenic epitopes that may serve as candidate immune epitopes in further studies. The study may serve to advance vaccine therapeutics-related research to better challenge XDR-*Alcaligenes faecalis-*associated infections.

## 2. Methodology

### 2.1. Proteome Subtraction

The whole proteome sequence (UniProt ID: UP000278916) of *A. faecalis* containing a total of 3667 proteins was downloaded from UniProtKB (UniProt, Geneva, Switzerland) [30]. The subtractive proteomics approach was then employed to shortlist target proteins and design epitope-based vaccines against *A. faecalis*. This was initiated with the screening of subcellular localization of each protein by utilizing the CELLO online tool (National Chiao Tung University, Hsinchu City, Taiwan) [31]. The server deploys an integrated SVM (support vector machine) system for predicting the localization of a specific protein in the cell. This was followed by subjecting the shortlisted extra-cellular proteins towards the utility of the BLASTp tool (NCBI, Bethesda, MD, USA) with default parameters to remove any human (Homosapiens, ID:9606) homologous proteins in a further target prioritization process [32]. Moreover, the overlapping sequences in the whole proteome were also assessed with the utility of the Cluster Data Base with High Tolerance (CD-HIT) suite [33]. This exclusion of any overlapping protein sequences was performed based on the ability of the improved clustering algorithm (cutoff value 0.8) to identify duplicated proteins having a shared identity of about 80% or more [34]. Finally, only highly antigenic and non-allergenic target proteins were identified amongst the shortlisted proteins by utilizing VaxiJen (Jenner Institute, Oxford, UK) [35] and Algpred2 (IIIT, New Delhi, India) [36], respectively. After the extensive analysis, only the shortlisted three target proteins were subjected to putative epitope predictions in the next step of the performed investigations.

### 2.2. Putative Immune Epitope Predictions in the Target Proteins

Screening of putative immune epitope predictions is a prerequisite in computational vaccinology approaches. In this study, an efficient and widely used web server, NetCTL 1.2 (DTU Health Tech, Lyngby, Denmark) [37], was deployed to predict T-cell (also called cytotoxic T lymphocytes) epitopes in the target protein sequences. The server used a trained model based on 886 known MHC class I ligands and 12 super-types to predict T-cell epitopes and classify on the basis of TAP (Transport Associated with Antigen Processing) and several other parameters. Similarly, the IEDB online server was used to predict HTL (helper T lymphocytes) epitopes in the target sequences by using a set of seven reference (human HLAs) alleles [38]. These predictions were mainly based on the binding affinity calculations and were depicted in percentile ranks to characterize HTL epitopes. Herein, a lower percentile rank for an HTL epitope was indicative of its higher binding affinity towards human HLAs. Additionally, all the shortlisted epitopes were also analyzed for interferon-gamma induction potential by utilizing the IFN-epitope server [39] to choose the best HTL epitopes. These investigations involved server-integrated algorithms to classify epitope sequences on the basis of obtained SVM scores and to predict IFN (interferon) response (inducing or non-inducing) for each HTL epitope [39]. Finally, B-cell epitopes were also predicted by using the ABCPred online server [40] with high accuracy on the basis of comparative analysis of input sequences with experimentally known B-cell epitopes [41]. After evaluations of antigenic and allergenic potential based on an integrated threshold scoring system, only shortlisted epitopes (T-cell, B-cell, and HTL) were used in the target-specific multi-epitope-based vaccine constructs presented in this study.

### 2.3. Target-Specific Multi-Epitope-Based Vaccine Designs against A. faecalis

The target-specific multi-epitope-based vaccine (MEVC) design process was performed on the basis of joining highly antigenic and non-allergenic putative immune epitopes. This included the addition of two epitopes from each class (T-cell, B-Cell, and HTL), along with an N terminal-linked hBD-2 adjuvant. Moreover, several linkers were used (EAAK, AAY, GPGPG, and KK) [26,42,43] to prevent self-folding and differentiation and to maintain the immunogenic ability of each epitope [44]. After topographical organization and assembly of each MEVC, the Robetta server [45] was deployed in 3D structural modelling of the vaccine constructs. The input sequence of amino acids was processed by the server with the integrated RoseTTAFold or comparative modeling approach to model (3D) structures of the vaccine constructs. This was followed by validations of accurate structural models for each vaccine by deploying the proSA-web [46] server reflected by the obtained Z-score, where deviations in the Z-score indicate errors in the tertiary structure of modeled proteins. Moreover, the PROCHECK [47] server was also deployed to assess the stereo-chemical properties and geometry through Ramachandran plot analysis of the modelled 3D structure. Additionally, each vaccine construct was also evaluated for several physiochemical properties by using the ProtParam online tool (Swiss Institute of Bioinformatics, Lausanne, Switzerland) [48] to depict molecular weight, theoretical PI, and other important parameters for each of the vaccine construct.

### 2.4. Molecular Docking and Interaction Analysis

The validated structural models for each of the vaccine construct were also subjected to molecular docking analysis with human TLR2 (Toll-like receptor-2) to depict the binding patterns [49]. This analysis was performed with the utility of HawkDock server with the deployed hybrid docking method providing several types of information, including the binding interactions and involved residues [50]. Additionally, it also offers the service of integrated MM-GBSA analysis for calculating the binding free energy of the docked complex. The retrieved human TLR-2 structure used in the docking analysis with each 3D vaccine model was downloaded from RCSB (Accession ID: 6nig). All the structures, including human TLR-2, were visualized and prepared in PyMOL software (Schrödinger, New York, NY, USA) before initiation of the molecular docking analysis with vaccine constructs.

### 2.5. In Silico Cloning of the MEVCs (Multi-Epitope-Based Vaccine Constructs)

The DNA sequence obtained after reverse translation of the vaccine construct sequences was also deployed in several in silico-based cloning approaches for computationally designed vaccine constructs [51,52]. This was achieved with the utility of the Java codon adaptation tool (JCat Tool, Technical University of Braunschweig, Braunschweig, Germany) for predicting the optimal expression ability of the epitope-based vaccine constructs in the widely used expression of host *E. coli* [53]. In this analysis, after choosing the appropriate host (*E. coli* strain K-12), the vaccine construct sequence (DNA) was analyzed for the percentage of GC content and Codon Adaptation Index Score to acquire the optimized DNA sequence for in silico cloning. Finally, the optimized DNA sequence was inserted into the pET-28a (+) expression vector with restriction enzyme sites (XhoI and EcoRI), and the plasmid maps were obtained using SnapGene software (SnapGene, San Diego, CA, USA).

### 2.6. Immune Simulation

The depiction of potential immune response for the proposed target-specific epitope-based vaccines was performed utilizing the online immune simulation tool C-ImmSim (National Research Council, Italy, Rome) [54]. The antigenic constructs were subjected to antibody production analysis to decipher the putative immune responses produced against vaccine constructs. This server deploys scoring matrix PSSM-based systems and machine learning modules to estimate the potential immune response against antigenic constructs. These evaluations include the production of an antibodies count on an arbitrary scale (xx/mL), while interferons and cytokines production are quantified in ng/mL against the antigenic constructs [55]. This approach is widely used in computational vaccinology approaches to suggest the experimental feasibility of epitope-based vaccine constructs against several pathogens.

## 3. Results

### 3.1. Target Proteins Annotation for A. faecalis

**Figure 1:**
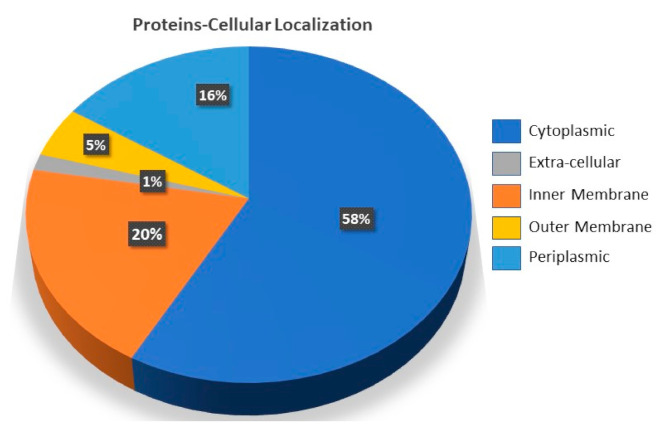
The distribution percentage of each protein based on cellular localization. The different colors represent the percentage of each type of localized proteins in the whole proteome sequence of *A. faecalis*.

Protein target annotation is a prerequisite step followed in the process of designing therapeutics [56,57]. This also involves identification of novel targets that can be used in vaccine design approaches [58]. Herein, the whole proteome sequences were first subjected to the pipeline of targets prioritization and characterization among the total of 6337 proteins of *A. faecalis*. This was achieved with the initial analysis of identifying proteins localization through the use of the online available server “CELLO”. This investigation helped to classify the ratio of proteins based on the putative localization in the cell. The analysis revealed different percentages of proteins localized in different parts of the cell, including the outer membrane, inner membrane, and extracellular proteins, as shown in Figure 1.

After this initial analysis, a total of 58 extracellular proteins were shortlisted as putative targets [59,60] for use in further demonstrations. This was followed by target annotation through subjecting the shortlisted proteins into analysis using the BLASTp tool. This examination resulted in the removal of nine protein sequences with a higher shared homology in the human genome. The remaining 49 putative target proteins were also analyzed through using the CD-HIT tool (Weizhong Li’s Group, Footscray, Australia), with no paralogs found. Moreover, the target proteins were further screened in antigenicity and allergenicity status evaluations. This helped to further remove the non-antigenic proteins and resulted in a total 45 proteins with a positive antigenicity status. Finally, these 45 proteins were then evaluated for allergenicity status, which revealed only 24 putative targets for a vaccine designed against *Alcaligenes faecalis.* The pipeline followed helped to characterize only three highly antigenic target proteins with the highest antigenicity scores >0.8. The three shortlisted proteins as putative targets used in further analysis are given in Table 1.

### 3.2. Immunogenic Epitopes Prediction for Target Proteins

Next, the putative target proteins sequences were subjected to prediction of T-cell epitopes, which are necessary antigen-related factors required for identification through MHC-I molecules in production of protective immune response [61]. During the initial screen, a total 45 for the target protein (A0A3G6HQC3), while 4 of each putative T-cell epitope were found for the targets A0A2U2BJQ9 and A0A3G6HK40. However, after evaluation of antigenicity and allergenicity status of each T-cell epitope for all the target proteins, all four T-cell epitopes shortlisted for target A0A3G6HK40 were found with allergenic status (Table S1). Hence, only two target proteins—A0A3G6HQC3 and A0A2U2BJQ9—were further processed with demonstrated non-allergenic potential of shortlisted T-cell epitopes. This analysis was followed by computational screening of putative B-cell and HTL epitopes for the two target proteins of the *Alcaligenes faecalis* proteome.

The highly immunogenic epitopes based on higher antigenicity score and non-allergenic status used in the design of putative vaccines are shown in Table 2.

### 3.3. Target-Specific Design of Peptide-Based MEVCs

**Figure 2:**
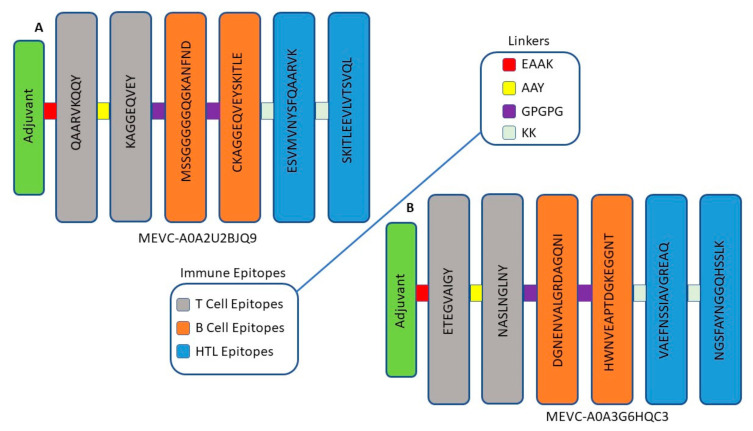
The topographical organization of each MEVC designed against the two target proteins. The different colors represent each type of epitope and linker used in the vaccine design procedure. Figure (**A**) represents the topographical organization of MEVC named (MEVC-A0A2U2BJQ9), while (**B**) represents the topographical organization of MEVC named (MEVC-A0A3G6HQC3) designed against each protein, respectively.

Bioinformatic analysis greatly helps in target identification, which leads towards designing therapeutics against human pathogens. Several available online platforms have been recently developed based on these in silico approaches [43,62]. The whole proteome-based therapeutics target mining and shortlisting of highly immunogenic epitopes was continued to design peptide-based vaccines against the two target proteins of *Alcaligenes faecalis*. This was achieved by the joining of the shortlisted epitopes with the help of different peptide linkers in the final vaccine designs. The topographical organization of each MEVC designed against the two target proteins is shown in Figure 2.

These peptide-based target-specific vaccine constructs against *Alcaligenes faecalis* of a length of 165 amino acids each were also evaluated for several physiochemical properties. Additionally, the antigenicity and allergenicity status of each full-length vaccine construct was also evaluated to confirm its validity in experimental processing, as shown in Table 3.

### 3.4. Modelling and Validation of Target-Specific Peptide-Based MEVCs

**Figure 3:**
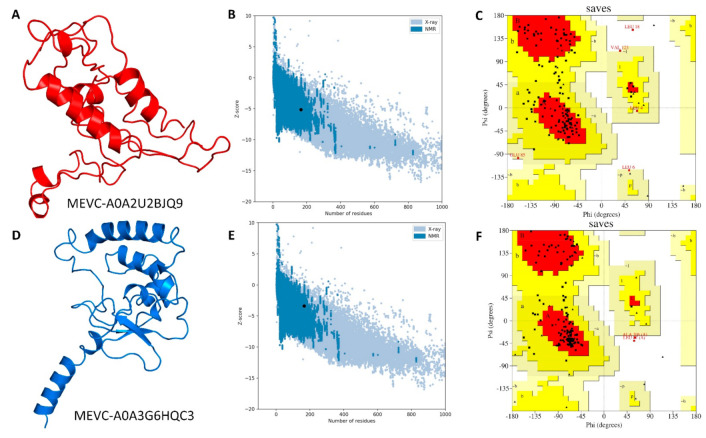
The modelled structures and validation analysis of the target-specific MEVC designs against *Alcaligenes faecalis.* Figures (**A**–**C**) represent the 3D modelled structure, Z-score graph, and Ramachandran plot for the vaccine construct (MEVC-A0A2U2BJQ9), while (**D**–**F**) show the 3D modelled structure, Z-score graph, and Ramachandran plot for the vaccine construct (MEVC-A0A3G6HQC3).

The target specific design of peptide-based MEVCs was followed by modeling the full length (165 amino acids) MEVCs, which included six highly antigenic epitopes (T-cell, B-cell, and HTL) for each protein. This was achieved through subjecting the vaccine construct sequences to structural modeling and validation analysis. Herein, the final MEVC-modeled structure after utilization of Robetta server is shown in (Figure 3A,D). The predicted (3D) model after confirmed visualization in PyMOL software was also subjected to validations of the modeled structure. This was performed by utilizing ProSA-web and PROCHECK servers [46,63]. Herein, the ProSA-web analysis revealed predicted Z-scores of −5.16 and −3.39 (Figure 3B,E), while PROCHECK resulted in a Ramachandran plot showing 70–80% of the residues in the most favored regions and only 1.6% of residues in the disallowed region for each MEVC, respectively (Figure 3C,F).

### 3.5. Molecular Docking and Interaction Analysis of the Designed MEVCs with Human TLR2

**Figure 4:**
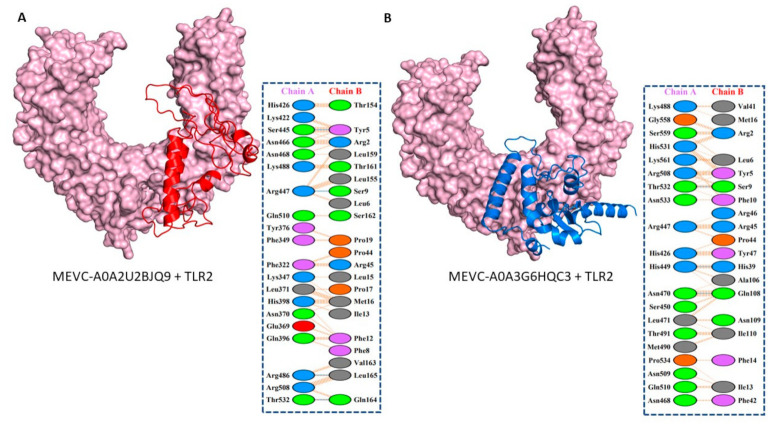
Demonstrates the docking complex and interaction patterns of each target-specific MEVC (red and blue), with the structure in the surface view (light pink color) represented as human TLR-2. Figure (**A**) shows the structural docking complex of MEVC-A0A2U2BJQ9 (red color) with human TLR-2 and the interaction patterns, while (**B**) shows the structural docking complex of MEVC-A0A3G6HQC3 (blue color) with human TLR-2 and the depicted interaction patterns.

Next, molecular docking evaluations were also performed for each of the two designed MEVCs with human TLR-2. Herein, the HDOCK server was employed to attain the docking complexes, and the best ones with the highest binding energy scores were further evaluated to depict the molecular interactions by utilizing PDBsum for the MEVC-A0A2U2BJQ9 + TLR2 complex (Figure 4A). Similar analysis was performed for the MEVC-A0A3G6HQC3 + TLR2 complex (Figure 4B), and several binding interactions including hydrogen bonds, salt bridges, and non-bonding contacts were depicted for each of the docking complexes. The analysis for the MEVC-A0A2U2BJQ9 + TLR2 complex with a binding energy score of −31.95 (kcal/mol) revealed the formation of four hydrogen bonds and 160 non-bonded contacts. Similarly, the MEVC-A0A3G6HQC3 + TLR2 complex having a binding energy score of −36.81 (kcal/mol) demonstrated the formation of three hydrogen bonds and 195 non-bonded contacts.

Furthermore, the MM-GBSA analysis was also performed for both of the docking complexes to curate individual binding free energies, as reported in previous studies [64,65,66,67]. This included depiction of van der Waals energy, electrostatic energy, Gibbs free energy, surface area, and the total binding energy to evaluate the interaction capacity of the two MEVCs with human TLR-2, as shown in Table S2.

### 3.6. In Silico Cloning Design of WP-MEVC

**Figure 5:**
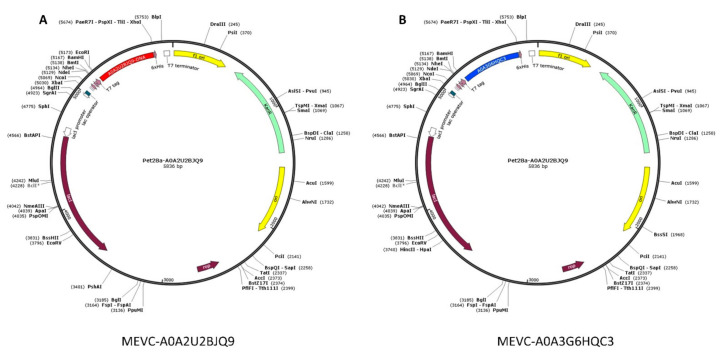
The in silico cloning designs (plasmid maps) in the Pet-28 expression vector using ECORI and XhoI restriction sites for the MEVCs (DNA sequence) against target proteins of *Alcaligenes faecalis*. Figure (**A**) shows the in silico cloning design for MEVC-A0A2U2BJQ9 (red color) in the Pet28 vector, while (**B**) shows the in silico cloning design for MEVC-A0A3G6HQC3 (blue color) in the Pet28 vector.

Furthermore, by utilizing the JCat server, the epitope-based vaccine designs were also subjected to a process for obtaining in silico clones for each MEVC DNA sequence in expression vector Pet28 used in *E. coli* strains. This was performed after obtaining the optimal Codon Adaptation Index scores of 1 and GC content of >50 percent for both of the vaccine sequences (DNA). These observations also suggested the probability of optimal expression of each vaccine construct in *E. coli* strains. The optimized DNA sequence for each of the vaccine constructs was then cloned into the expression pET28a (+) vector using restriction enzyme (XhoI and ECORI) sites. Finally, the plasmid maps with inserted optimized (DNA) sequences were then created using Snapgene software, as shown in Figure 5.

### 3.7. Immune Simulation of the Proposed Vaccine

**Figure 6:**
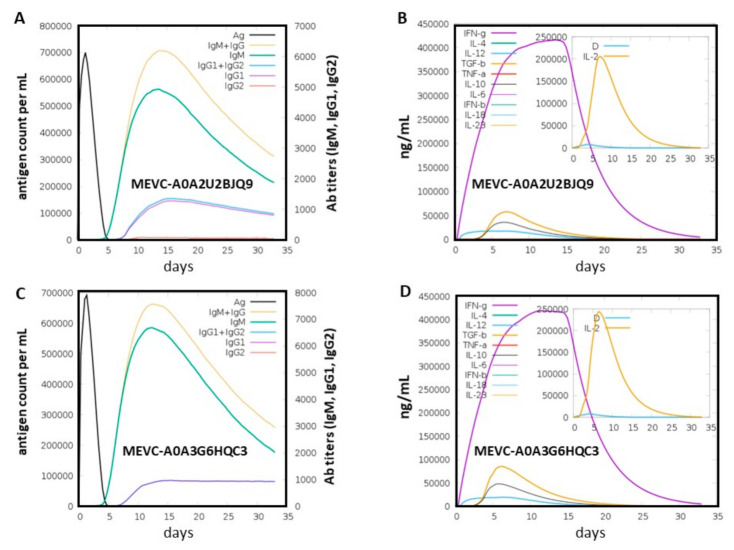
The evaluation of highly antigenic epitope-based vaccine designs against the two target proteins of *Alcaligenes faecalis*. Figures (**A**,**B**) show the immune simulation graphs obtained for MEVC-A0A2U2BJQ9, showing the putative antigen count/mL/day plotted against Ab titers and also cytokines and interleukins production against the target protein. Similarly, figures (**C**,**D**) show the immune simulation graphs obtained for MEVC-A0A3G6HQC3 representing the putative immune response in the form of produced Ab titers, cytokines, and IFN responses against the protein target-specific vaccine construct.

Finally, the highly antigenic MEVCs were also assessed for induction of potential immune responses through an in silico immune simulation approach. This analysis involved the quantification of putative antibody titers produced for each of the target-specific vaccine constructs against *Alcaligenes faecalis*. This revealed higher antigenic counts (>600,000) found for both (MEVC-A0A2U2BJQ9 and MEVC-A0A3G6HQC3) of the vaccine constructs on day 2, followed by complete neutralization of the antigen and initiation of antibody production on day 5. It was predicted that production of antibodies (IgM and IgM1 + IgM2) starting from day 5 might be as high as 6000–7000 xx/mL between day 10 and day 15 for both of the vaccine constructs (Figure 6A,C). The highest peaks among the antibody response produced against the antigenic vaccine constructs included an elevated trend in the production of IgM + IgG antibodies with the highest peaks. This was followed by higher peaks of immunoglobulins (IgM, IgG1, and IgG2) produced during the time period of 10–15 days. Additionally, the immune response induction potential was also evaluated through putative production (ng/mL) of interferon (IFN), interleukins (IL), transforming growth factors (TGF), and transforming necrosis factor (TNF) for each of the MEVCs. Immune response was evident, with the highest peaks (>400,000) observed during days 10 to 15, with IFN-g production against both of the MEVCs (Figure 6B,D) designed in this study. Similarly, the different concentrations obtained for several cytokines and interleukins produced against each MEVC were also observed. However, further analysis using advanced experimental techniques (in vitro and in vivo) are required to depict the potential ability of the designed MEVCs in the production of adaptive immunity against *Alcaligenes faecalis*.

### 3.8. Limitations of the Study

In this study, whole proteome-based target-specific vaccine designs were explored. The several antigenic, allergenic, and physiochemical properties were also explored for each designed vaccine candidate. Nonetheless, further experimental validations of the in silico-based studies’ final vaccine constructs are needed. The important experimental procedures followed in synthetic vaccine design approaches, including expression and purification of the MEVCs, may aid in the further advancement of these candidate vaccines as potential therapeutics against *A. faecalis*.

## 4. Conclusions

Currently, in silico methods for the identification of expressed proteins and promiscuous antigens and peptides of pathogen are being highly used in vaccine development. Additionally, in silico vaccine design methods have been validated using a wide range of in vivo and in vitro experiments, showing promising results. These methods are not only efficient in vaccine development time and cost but also reduce the labor input. The current study depicts whole proteome-based target annotation with two shortlisted putative protein targets in vaccines designed against *A. faecalis*. It also demonstrates the target protein-specific prediction of numerous immune epitopes (including T-cell, B-cell, and HTL). Furthermore, topographical arrangement, 3D modeling, structural validation, and physiochemical properties evaluations of the MEVCs were performed. Moreover, the ability of potential interactions between the MEVCs (MEVC-A0A2U2BJQ9 and MEVC-A0A3G6HQC3) and human TLR-2 were also confirmed through molecular docking approaches that demonstrated strong hydrogen bonding interactions. The designed MEVCs were also subjected to the utility of in silico cloning and immune simulation approaches. This demonstrated the highly related immune response induction potential for both of the vaccine constructs presented in this study. Nevertheless, further experimental validation is required to verify the potential utility of these MEVCs as therapeutic options against *Alcaligenes faecalis*.

## Figures and Tables

**Table 1:** The three prioritized target proteins with demonstrated highest antigenicity scores.

Accession ID (UniProt)	Prioritized Proteins	Antigenicity Scores	Allergenicity Status
A0A2U2BJQ9	Hcp1 family type VI secretion system effector	1	Non-Allergen
A0A3G6HQC3	Uncharacterized protein	0.93	Non-Allergen
A0A3G6HK40	Uncharacterized protein	0.87	Non-Allergen

**Table 2:** Showing the antigenicity- and allergenicity-based shortlisted epitopes against each target protein of *Alcaligenes faecalis*.

Accession ID (UniProt)	Epitope	Number of Epitopes	Peptide Sequences	Antigenicity Scores	Allergenicity Status
A0A3G6HQC3	T-cell	2	QAARVKQQY KAGGEQVEY	0.93 0.75	Non-allergens
A0A3G6HQC3	B-cell	2	MSSGGGGGQGKANFND CKAGGEQVEYSKITLE	2.7 1.3	Non-allergens
A0A3G6HQC3	HTL	2	ESVMVNYSFQAARVK SKITLEEVLVTSVQL	0.8 0.5	Non-allergens
A0A2U2BJQ9	T-cell	2	ETEGVAIGY NASLNGLNY	0.95 0.89	Non-allergen
A0A2U2BJQ9	B-cell	2	DGNENVALGRDAGQNI HWNVEAPTDGKEGGNT	0.5 2.2	Non-allergens
A0A2U2BJQ9	HTL	2	VAEFNSSIAVGREAQ NGSFAYNGGQHSSLK	0.5 0.79	Non-allergens

**Table 3:** The individually evaluated physiochemical properties, antigenicity, and allergenicity status of each target-specific peptide-based vaccine design against *Alcaligenes faecalis*.

**Vaccine Name**	Peptide Sequences	Amino Acids (Number)	Molecular Weight (kd)	Theoretical pI	Aliphatic Index	Hydropathicity (GRAVY)	Antigenicity Score	Antigenicity Status	Allergenicity
MEVC-A0A2U2BJQ9	MRVLYLLFSFLFIFLMPLPGVFGGIGDPVTCLKSGAICHPVFCPRRKQIGTCGLPGTKCCKKPEAAKQAARVKQQYAAYKAGGEQVEY GPGPGMSSGGGGGQGKANFND GPGPGCKAGGEQVEYSKITLEKKESVMVNYSFQAARVKKKSKITLEEVLVTSVQL	165	17.5 kd	9.41	72.67	−0.145	1.16	Antigen	Non-Allergen
MEVC-A0A3G6HQC3	MRVLYLLFSFLFIFLMPLPGVFGGIGDPVTCLKSGAICHPVFCPRRYKQIGTCGLPGTKCCKKPEAAKETEGVAIGYAAYNASLNGLNYPGPGDGNENVALGRDAGQNIGPGPGHWNVEAPTDGKEGGNTKKVAEFNSSIAVGREAQKKNGSFAYNGGQHSSLK	165	17.2 kd	8.91	69.21	−0.251	0.97	Antigen	Non-Allergen

## Data Availability

The data presented in this study are available within the article.

## Supplementary Materials

The following supporting information can be downloaded at: https://www.mdpi.com/article/10.3390/vaccines10030462/s1, Table S1: Showing the antigenicity and allergenicity status for each of the shortlisted T-cell epitopes against the target protein of (Accession ID:A0A3G6HK40) of *Alcaligenes faecalis,* Table S2: MM-GBSA analysis showing individual binding energies for each of the docking complexes.
