# Supplementary material for: Whole Proteome-Based Therapeutic Targets Annotation and Designing of Multi-Epitope-Based Vaccines against the Gram-Negative XDR-Alcaligenes faecalis Bacterium
Source: Vaccines (Basel). 2022 Mar 17;10(3):462. doi: 10.3390/vaccines10030462 (PMC8955209; doi:10.3390/vaccines10030462)
Supplement: Supplementary file 1 [file vaccines-10-00462-s001.zip › vaccines-1602273-supplementary.pdf]

Table S1: showing the antigenicity and allergenicity status for each of the shortlisted T cell epitope against target protein of (Accession ID:A0A3G6HK40) of *Alcaligenes faecalis.*

Accession ID (Uniprot)	Epitope	Peptide sequences	Antigenicity Scores	Allergenicity Status
**A0A3G6HK40**	T cell	ITDGWELAV	1.5	Allergen
**A0A3G6HK40**	T cell	RTEQGQHLV	0.6	Allergen
**A0A3G6HK40**	T cell	NTATITNQL	0.5	Allergen
**A0A3G6HK40**	T cell	LTATGNSSL	0.7	Allergen

Table S2: MM-GBSA analysis showing individual binding energies for each of the docking complex.

Docking Complex	Van der Waals energy (VDW)	Electro static energy (ELE)	Gibbs free energy (GB)	Surface Area (SA)	Total binding energy
**MEVC-A0A2U2BJQ9**	-118.67	-545.86	647.66	-15.09	-31.95
**MEVC-A0A3G6HQC3**	-119.17	-552.95	650.35	-15.05	-36.81
